# Mutation analysis of genes in the *EGFR* pathway in Head and Neck cancer patients: implications for anti-EGFR treatment response

**DOI:** 10.1186/1756-0500-7-337

**Published:** 2014-06-04

**Authors:** Carolien Boeckx, Christine Weyn, Isabelle Vanden Bempt, Vanessa Deschoolmeester, An Wouters, Pol Specenier, Carl Van Laer, Danielle Van den Weyngaert, Mark Kockx, Jan B Vermorken, Marc Peeters, Patrick Pauwels, Filip Lardon, Marc Baay

**Affiliations:** 1 Center for Oncological Research (CORE) Antwerp, Laboratory of Cancer Research and Clinical Oncology, University of Antwerp, Wilrijk, Belgium; 2 HistoGeneX, Berchem, Belgium; 3 Department of Medical Oncology, Antwerp University Hospital, Edegem, Belgium; 4 Department of Otolaryngology, Antwerp University Hospital, Edegem, Belgium; 5 Department of Radiation Therapy, Antwerp University Hospital, Edegem, Belgium; 6 Department of Pathology, Antwerp University Hospital, Edegem, Belgium

**Keywords:** Head neck squamous cell carcinoma, EGFR inhibitors, Cetuximab, Resistance, KRAS mutations, EGFRvIII mutations, EGFR tyrosine kinase mutations, HPV

## Abstract

**Background:**

Targeted therapy against the Epidermal Growth Factor Receptor (EGFR) is among the most promising molecular therapeutics for Head and Neck Squamous Cell Carcinoma (HNSCC). However, drug resistance limits the clinical efficacy of anti-EGFR monoclonal antibodies and no predictive biomarker has entered the clinic yet.

**Methods:**

A retrospective clinical study was performed utilizing pathological specimens from 52 newly diagnosed HNSCC patients. These patients were screened for mutations in *EGFR* and *KRAS*. Tyrosine kinase mutations in *EGFR* and *KRAS* mutations were evaluated by high resolution melting analysis (HRMA), whereas *EGFRvIII* was determined using one-step real-time PCR. Finally, patient samples were screened for HPV-DNA by GP5+/6+ PCR. Survival analysis was performed using Kaplan-Meier analysis and significance was calculated using log-rank statistic.

**Results:**

In our study population no EGFRvIII mutations were present. However, two silent mutations were found; T785T in exon 20 and R836R in exon 21 of the *EGFR* gene. Additionally, HRMA revealed an abnormal *KRAS* melting pattern in 7.0% of the samples. However, the KRAS StripAssay could confirm only one sample with a G12S mutation and none of these samples could be confirmed by direct sequencing. HPV DNA was present in 3/25 larynx and 9/27 oropharynx tumors.

**Conclusion:**

The low rate of *EGFR* and *KRAS* mutations in this Belgian HNSCC population suggests that these genes will probably not play a major role in predicting response to anti-EGFR therapy in HNSCC. Hence, other predictive markers need to be discovered in order to optimize EGFR targeting therapy.

## Background

Head and neck squamous cell carcinoma (HNSCC) is a heterogeneous and complex disease, having a severe impact on the quality of life of patients and survivors [1]. At time of diagnosis, 60 to 70% of patients present with advanced disease, affecting the survival of these patients negatively [2]. In up to 90% of HNSCC tumors, increased expression of the epidermal growth factor receptor (EGFR) is observed [3-5]. Increased EGFR expression is associated with advanced stage of the disease, poor survival and resistance to chemo- and radiotherapy [3,6]. In only 50% of HNSCC patients, the current conventional treatment strategies, including surgery, chemotherapy and radiation, are effective, underscoring the need for new approaches to treat this malignancy [7,8]. Moreover, the existing cytotoxic therapies are non-selective and associated with considerable toxicity. Therefore, personalized medicine using targeted therapies will hopefully achieve the much-needed progress in HNSCC treatment.

As EGFR signaling plays an important role in the development and progression of HNSCC, it is one of the most promising targets for molecular targeted therapies [3-5]. The addition of cetuximab to platinum-fluorouracil chemotherapy, for instance, improved overall survival when given as first-line treatment in patients with recurrent or metastatic HNSCC [9]. However, resistance to EGFR inhibitors presents a therapeutic challenge, since intrinsic as well as acquired resistance after initial response has been described frequently [10]. Therefore, it is important to understand the mechanism of intrinsic resistance, as a further selection of patients who might benefit from the addition of cetuximab will increase its beneficial effect and most likely its cost-effectiveness.

In other forms of cancer where anti-EGFR therapy is used, molecular profiling of the tumor has become essential for customized medical treatment decision. *EGFR* and *KRAS* mutations strongly influence the benefit of treatment with anti-EGFR therapies, such as panitumumab and cetuximab used for treating metastatic colorectal cancer (mCRC) and tyrosine kinase inhibitors (TKIs) used for advanced non-small cell lung cancer (NSCLC) [11-14]. Although several reports have described *EGFR* and *KRAS* mutations in HNSCC, the mutation frequency can differ among ethnic groups. In the present study we aimed to correct for the heterogeneous nature of this disease by including only HNSCC patients eligible for cetuximab therapy and suffering from two common subtypes of HNSCC (oropharynx and larynx). Moreover, since infection with human papillomavirus (HPV) is a driver of HNSCC carcinogenesis, and HPV-positive cancers form a distinct group with better clinical outcome within HNSCC [15,16], we determined the presence of HPV infection as well.

## Methods

### Patient samples

Formalin fixed, paraffin-embedded (FFPE) tissues from 52 newly diagnosed HNSCC patient samples (27 oropharyngeal and 25 laryngeal SCC) were retrieved from the department of Pathology, Antwerp University Hospital (Edegem, Belgium). The tissues were obtained from biopsies or resections performed between 2002 and 2010. At that time, all patients agreed that residual material from resections was used for studies (consented at the time of hospitalization); no recurrent approval from the local ethic committee was required. As EGFR targeted therapies are approved in patients with locally advanced or recurrent/metastatic disease, our patients were selected for stage III or higher to most accurately resemble this population.

### Screening of *EGFR* tyrosine kinase and *KRAS* mutations

Prior to DNA extraction, tumor tissue was enriched by manual macrodissection and isolated using the QIAamp® DNA FFPE Tissue isolation kit (Qiagen, Venlo, The Netherlands). Concentration and purity were defined using the Nanodrop ND-1000 spectrophotometer (Isogen, Sint-Pieters-Leeuw, Belgium).

Genomic DNA from patients was used for mutation analysis in the *KRAS* gene and *EGFR* tyrosine kinase domain by high resolution melting analysis (HRMA). Screening of mutations in exon 2 (codon 12 and 13) of the *KRAS* gene was performed as described previously [17]. Patient samples with an abnormal melting pattern were subjected to KRAS StripAssay® (ViennaLab Diagnostics GmbH, Vienna, Austria), according to the manufacturer’s recommendation. This assay screens for 10 *KRAS* mutations in codon 12 and 13 of exon 2.

Additional DNA was isolated from patients suspected of *KRAS* mutations, amplified by PCR and sequenced using Sanger sequencing. Primers for the 178-bp amplicon of exon 2 were 5’-GTAAAACGACGGCCAGGTGTGACATGTTCTAATATAG-3’ (forward) and 5’-TTGGATCATATTCGTCCACAA-3’ (reverse). PCR reaction was performed in 25 μl reaction containing 1× buffer, 1 mM MgCl_2_, 0.5 μM of each primer, 0.25 mM dNTPs, 1 μl Taq polymerase and 5 μl genomic DNA (10 ng/μl). PCR cycling was performed on a Px2 Thermal Cycler (Thermo Electron Corporation) and was run as follows: 15 minutes at 95°C and 35 cycles of 60 seconds at 94°C, 60 seconds at 55°C and 60 seconds at 72°C, followed by 10 minutes at 72°C. This PCR product was purified using ExoSAP-IT (Affymetrix, Cleveland, OH, USA) and for validation, this product was used as template for direct sequencing with the Big Dye Terminator v1.1 kit (Applied Biosystems, Foster City, CA, USA) using M13tag primers (Eurogentec, Seraing, Belgium). The reaction mixture consisted of 1.1× sequencing buffer, 0.2 μl Big Dye mix, 625 nM M13tag primer 5’-GTAAAACGACGGCCAG-3’ and 1 μl of purified template in a total volume of 4 μl. The reaction was run on a Rapid Cycler Instrument 2 (Idoha Technology Inc., Salt Lake City, UT, USA) according to the following protocol: initial denaturation at 95°C for 30 sec and 25 cycles in the following sequence: 96°C for 10 sec, 50°C for 5 sec and 60°C for 2 min. The sequencing reactions were run on a 3130 XL Genetic Analyzer (Applied Biosystems). Sequencing data were analyzed using SeqScanner software v1.0 (Applied Biosystems).

Primers and reaction conditions for detection of *EGFR* tyrosine kinase mutations in exon 19, 20 and 21 were adapted from Heideman et al. [18]. Amplification and generation of melting curves were performed on a Lightcycler® 480 (Roche Diagnostics Gmbh, Mannheim, Germany) and analyzed using GeneScanning software (Roche). HRMA products with a deviating *EGFR* melting pattern were directly purified using ExoSAP-IT (Affymetrix) and the purified PCR product was used as template for direct Sanger sequencing (Big Dye Terminator v1.1 kit (Applied Biosystems,)) using M13tag primers, as described above.

### EGFRvIII mutation detection using real-time PCR

The determination of EGFRvIII mRNA expression was performed by semi-quantitative real-time PCR. Total RNA was obtained from FFPE tumor tissue with the High Pure RNA Paraffin Isolation kit (Roche) according to the manufacturer’s recommendation. Prior to extraction, manual macrodissection was performed to enrich the sample for tumor cells.

After extraction, one-step real-time PCR was performed on the LightCycler 480 I instrument (Roche) using TaqMan RNA amplification kit (Roche). Thereby, 2 PCR reactions were performed, one for the specific amplification of EGFRvIII mRNA using a primer set targeting EGFR exon 1 and exon 8 (EGFRe1/8) and the other one for the amplification of total EGFR mRNA using a primer set targeting an unaffected region of the EGFR gene (exon 9 and 10, EGFRe9/10). The primer pairs are given in Table 1. For the detection of the EGFRvIII product, a fluorescently labeled oligonucleotide TaqMan probe (Eurogentec) was used. For the detection of total EGFR PCR product, YOPRO intercalating dye (Invitrogen, Merelbeke, Belgium) was used. Concentrated samples were diluted to a maximum of 20 ng/μl. The input varied between 18.5 ng and 100 ng. RNA template was added in a reaction mix consisting of 300 nM forward and reverse primer each, 3 mM Mn^2+^, 0.1 mM YoPro (EGFRe9/10 reaction) or 200 mM TaqMan probe (EGFRe1/8 reaction), 1× RNA mix (TaqMan RNA amplification kit, Roche) in a total volume of 25 μl. Samples were run in duplicate.

**Table 1:** Primers (Eurogentec) used for detection of EGFRvIII by real-time PCR

**Oligo name**	**Oligo type**	**Mod5’**	**Sequence**	**Mod3’**
**EGFRe1-F**	forward primer		GAGTCGGGCTCTGGAGGAA	
**EGFRe8-R**	reverse primer		GGCCCTTCGCACTTCTTACA	
**EGFRe1/8-TM**	TaqMan probe	6-FAM	AAAGGTAATTATGTGGTGACAGATCACGGCTC	BHQ-1
**EGFRe9/10-F**	forward primer		TCCTGCCGGTGGCATTT	
**EGFRe9-R**	reverse primer		TGTGGATCCAGAGGAGGAGTATG	

As positive control, mRNA derived from an EGFRvIII mutant glioblastoma cell line U87del (kind gift from dr. Furnari from the Ludwig Institute for Cancer Research, La Jolla, CA, USA) was used. The cycling conditions are shown in Table 2. Data were analyzed using the Lightcycler 480 software release 1.5.0 software (Roche).

**Table 2:** Cycling condition for one-step RT-PCR

**Stage**	**Temperature**	**Time**	**# cycles**
**Stage I preheating**	95°C	1 min	1
**Stage II: reverse transcription**	60°C	30 min	1
**Stage III: activation**	95°C	10 min	1
**Stage IV: cycling**	95°C	15 sec	45
	60°C	1 min	
**Stage V: denaturation**	95°C	1 min	
**Stage VI: melting**	50°C-85°C	Continues measuring	
**Stage VII: cool**	40°C	30 sec	

### HPV detection

HPV detection by GP5+/6+ PCR was carried out on all 52 HNSCC samples, as described previously [19]. Detection of PCR products was performed in an enzyme immunoassay format as described by Jacobs et al. [20].

### Statistical analysis

All experimental data were the combined result of at least two independent experiments. Prognostic relevance of HPV infection was assessed by survival analysis using the Kaplan–Meier method and survival curves were analyzed using the log-rank test. The index date for survival time calculation was defined as the date of diagnostic confirmation for HNSCC. The days of observation (overall survival time) were calculated from the index date to the date of last information/death. For progression free survival time, the days of observation were calculated from the index date to the first date of progression, death or the date of last information. All analyses were conducted using SPSS (version 20, SPSS Inc., Brussels, Belgium). Significance for all statistics was recorded if p < 0.05.

## Results

### Patient characteristics

Of the 52 HNSCC patients from whom tumor tissue could be obtained, most clinical data could be retrieved. Further details on these patients are summarized in Table 3.

**Table 3:** Clinical data from 52 HNSCC patients

	**Oropharynx**	**Larynx**	**Overall population**
**Patient characteristics**			
Total no. of patients	27	25	52
Median age (years)	62.75	62.17	62.58
**Sex**			
Male	21	21	42
Female	6	4	10
**Grade of differentiation**			
Good	4	7	11
Moderate	17	10	27
Poor	0	1	1
Unknown	6	7	13
**TNM**			
T1	2	0	2
T2	6	0	6
T3	5	10	15
T4	14	15	29
N0	5	12	14
N1	1	5	6
N2	17	6	23
N3	3	2	5
Nx	1	0	1
M0	21	22	43
M1	3	2	5
Mx	3	1	4
**Stage**			
III	3	8	11
Iva	19	13	32
Ivb	4	2	6
Ivc	1	2	3
**Smoking**			
Yes	9	6	15
Ex	4	6	10
No	1	0	1
Unknown	13	13	26
**Alcohol abuse**			
Yes	7	7	14
Ex	1	1	2
No	3	2	5
Unknown	16	15	31

### EGFR mutation analysis: EGFR tyrosine kinase and EGFRvIII mutation

**Figure 1:**
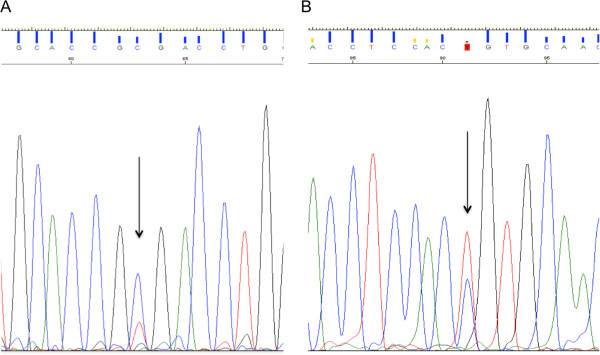
**Sequencing result of two HNSCC patients. A**: EGFR exon 20p results in a T > C mutation at nucleotide 2354 and **B**: EGFR exon 21 displays at nucleotide 2508 C > T. Both mutations result in a silent nucleotide polymorphism, T785T and R836R for exon 20 and exon 21 respectively.

HRMA was used as a first-line screening method for *EGFR* tyrosine kinase mutations. DNA samples yielding a sufficiently high DNA concentration and good purity were screened for mutations in exon 19 to 21 of the *EGFR* gene. In total, 46 HNSCC patient samples were available for *EGFR* mutation screening. Failure to generate a melting curve was present in 7, 7, 5 and 10 samples for exon 19, 20p, 20d and 21, respectively. HRMA could not detect any aberrant melting curves in exon 19, whereas in exons 20 and 21 abnormal melting curves were detected. Therefore, these samples were sequenced in order to confirm and determine the mutation. Although we did not detect any missense mutations, two patients were found with a silent mutation; T785T (exon 20p) and R836R (exon 21) (Figure 1A and 1B).

Furthermore, screening for *EGFRvIII* mutations was performed by one-step real-time reverse transcriptase PCR. Of the 52 HNSCC samples, 8 failed to amplify. In the remaining 44 HNSCC samples, no *EGFRvIII* mutations could be detected.

### *KRAS* mutation

**Figure 2:**
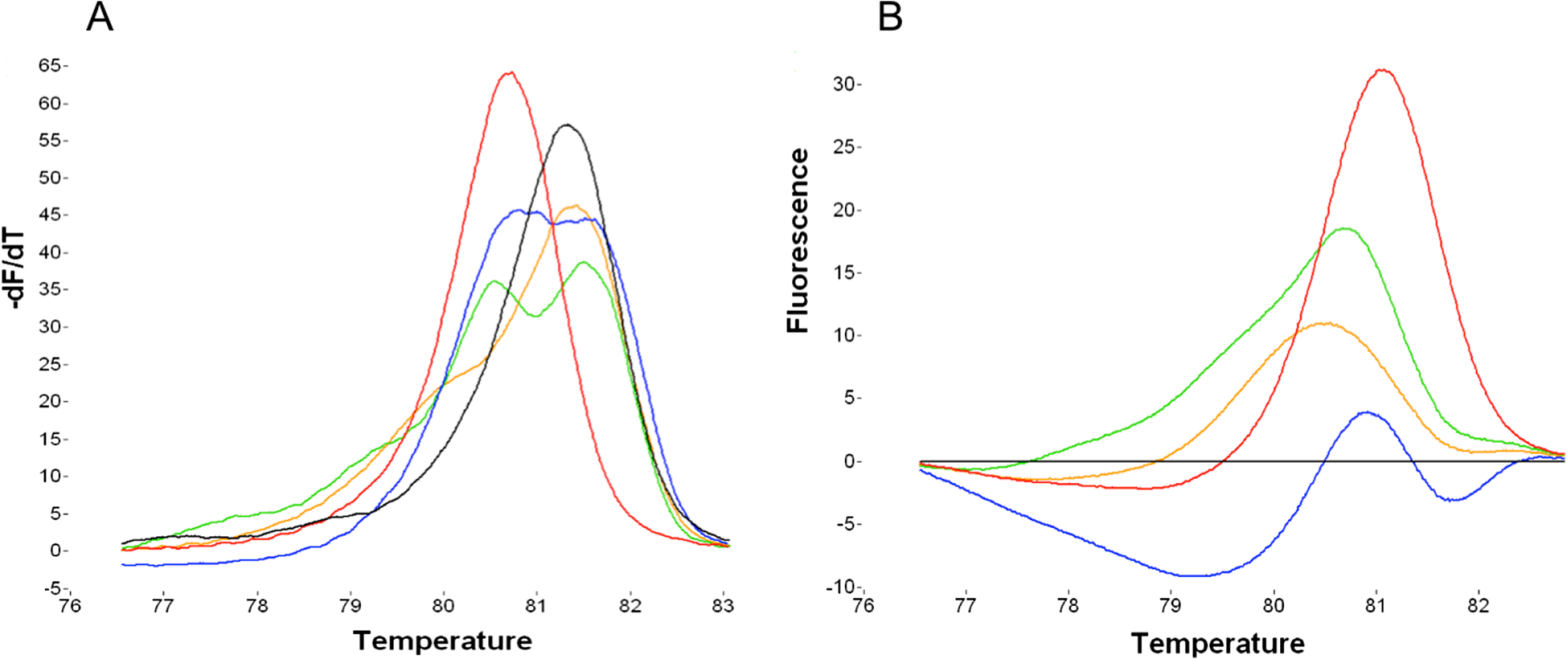
**Derivative plot (A) and difference plot (B) of the normalized high resolution melting curves of the three HNSCC samples suspected of *KRAS *mutation, together with two cell lines; A549 (homozygote G12S, red) and SC263 (homozygote wild type, black).** All samples show an abnormal derivative plot compared to the wild type SC263 plot.

Similar to the screening method of *EGFR* tyrosine kinase mutations, we screened for mutations in codon 12 and 13 of the *KRAS* gene using HRMA. Of the 52 HNSCC samples, five could not be used for screening due to reduced DNA concentration or insufficient purity of the DNA samples. Another 4 samples failed to generate a melting curve; hence no mutation status could be determined. Of the remaining 43 HNSCC patients, three samples presented with an aberrant melting profile, consistent with the positive control (*KRAS* mutated cell line), see Figure 2. The other 37 samples were wild type for codon 12 and 13 of the *KRAS* gene, as determined by HRMA.

For validation, these three samples were subjected to further analysis using the KRAS StripAssay®. These results showed a very weak band for the G12S mutations (green curve in Figure 2) for one sample, one other sample was wild type for 10 *KRAS* mutations (yellow curve in Figure 2) and one sample could not be evaluated due to failure of the test (blue curve in Figure 2). To further confirm these results, additional DNA was isolated for *KRAS* mutation screening by dideoxy sequencing for these three samples. Surprisingly, all samples showed the wild type GGTGGC sequence.

### HPV infection in oropharygeal and laryngeal cancer

**Figure 3:**
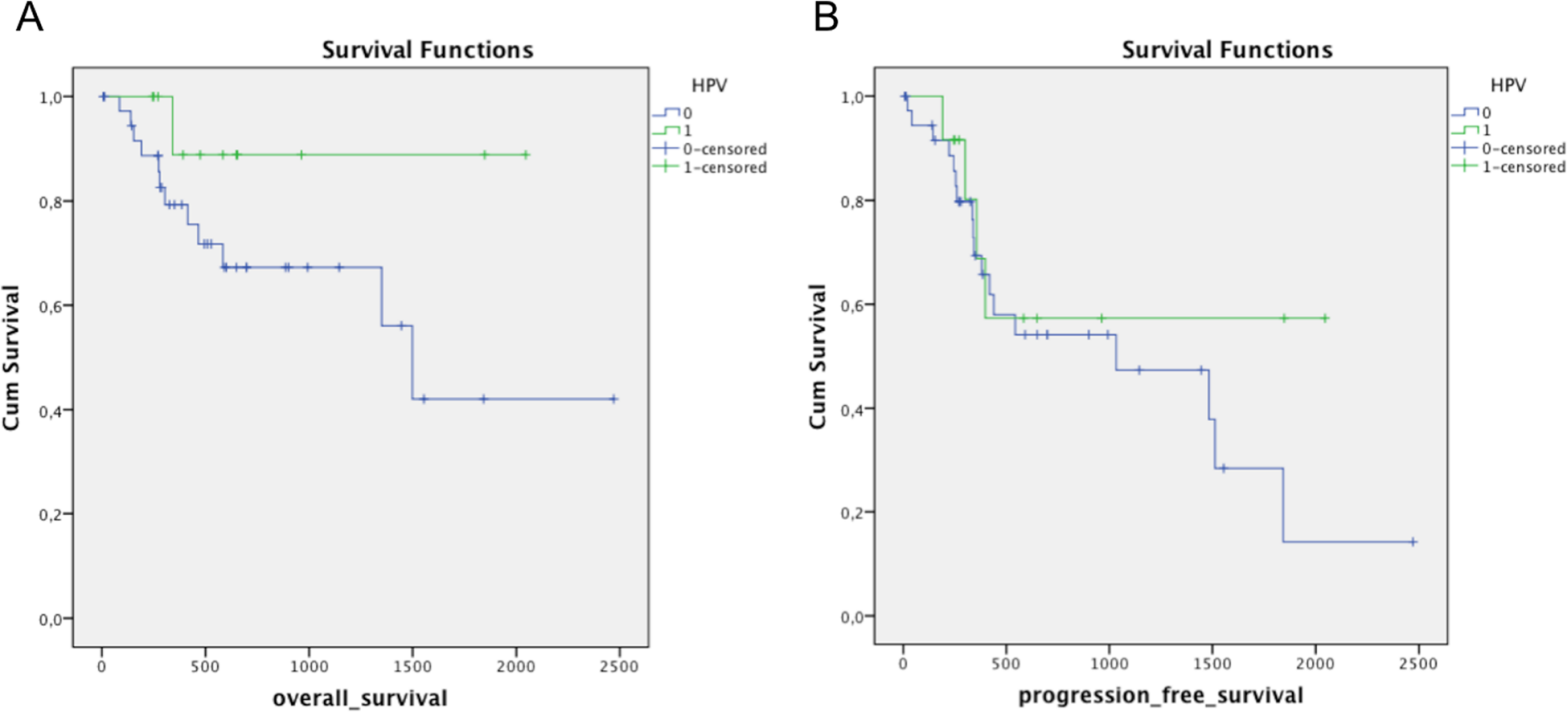
**Kaplan-Meier survival analysis for HPV infection in HNSCC for overall survival (A) and progression free survival (B).** Significance was calculated using log-rank statistic (p = 0.125 and p = 0.374, respectively).

Screening of our HNSCC patient population identified 12 out of 52 HPV positive HNSCC tumors (23.1%), nine tumors were located in the oropharynx (33.3%) and three in the larynx (12.0%).Statistical analysis did not show any significant survival benefit (overall survival and progression free survival) for patients with HPV-related HNSCC tumors, p = 0.125 and p = 0.374 respectively (Figure 3A and B).

## Discussion

As personalized cancer medicine – in particular drugs based on genetics – will become more and more important in the future, molecular profiling of the tumor prior to treatment becomes essential. Unfortunately, no predictive biomarker for the promising anti-EGFR therapeutics in the treatment of HNSCC has entered the clinic yet.

Pinpointing mechanisms of resistance to EGFR targeting agents in HNSCC has proven difficult due to several factors. First, HNSCC is a heterogeneous disease, affecting several anatomical structures located within the head and neck region. Second, mutation frequencies might vary between different ethnic groups. And finally, stages of tumors might be characterized by certain mutations. Therefore, we tried to correct for these factors by including only Caucasian laryngeal and oropharyngeal tumors eligible for cetuximab therapy and evaluated whether the mutation frequencies are in line with the frequencies reported in the literature. Furthermore, we determined the incidence of HPV in these 52 HNSCC patients as well.

Sustained EGFR signaling can be elicited at different levels in the signaling cascade, including at the level of the target itself. Compensatory mutations in the tyrosine kinase domain of *EGFR* or the constitutively active and truncated form of *EGFR* (i.e. EGFRvIII) are shown to predict response to anti-EGFR therapy in other tumor types [13,14,21,22]. We observed no *EGFR* tyrosine kinase missense mutations, although two silent mutations (T785T and R836R) were detected. These results indicate that *EGFR* tyrosine kinase mutations are uncommon in this Belgian HNSCC patient population. This is in concordance with literature where mutation frequencies vary between 0 and 8% [23-25]. On the other hand, Murray et al. detected an *EGFR* tyrosine kinase mutation frequency of 15.8%, albeit in a small sample population (n = 19) [5]. The EGFRvIII mutation has been reported previously in HNSCC by real-time PCR and immunohistochemistry. However, subsequent studies have provided conflicting evidence [11,16,26,27]. In these studies, the *EGFRvIII* mutation frequency in HNSCC varies between 0% and 42%. In our study population, no *EGFRvIII* mutations could be detected using one-step real-time PCR on FFPE material.

*KRAS* serves as a mediator between extracellular ligand binding and intracellular transduction of signals from EGFR to the nucleus. Therefore, aberrant expression of *KRAS* might result in sustained EGFR signaling despite inhibition. The *KRAS* mutation frequency in HNSCC observed in literature varies between 0 and 6% [23,26,28]. This study identified three out of 43 samples with an aberrant HRMA melting profile. Although these samples showed aberrant melting profiles, indicative of the presence of low level sequence variants, no mutations could be identified with Sanger sequencing and only one (G12S mutation) could be confirmed with the KRAS AssayStrip®. These conflicting results can be explained by two main factors, which influence test results. First, genetic heterozygosity within the tumor might result in mutation levels below the analytical sensitivity of dideoxy sequencing (10 - 30%) [17,29], whereas the detection limit of HRMA was previously reported at 3% [17] and the KRAS StripAssay® detect at least 1% mutated alleles. Second, the DNA used in this study was extracted from FFPE sections and formalin fixation has adverse effects on DNA. Consequently, Taq polymerase errors can cause PCR artifacts during amplification [29,30]. Therefore, positive samples were repeated by an independent round of DNA isolation and amplification, to avoid false positive results. However, it was not possible to make a distinction between low-level mutations and sequence artifacts by formalin fixation, as fresh frozen tissue from these patients was unfortunately not available.

As HPV positive HNSCC tumors tend to have a more favorable outcome, we screened our 52 patients for HPV DNA. In this Belgian study population, 33.3% of the oropharyngeal cancer and 12.0% of the laryngeal tumors contained HPV DNA. Frequencies reported in literature can range from 0% to 100% [31-33], depending on several factors, including differences in ethno-geographic regions, combination of different subtypes of HNSCC and differences in the applied HPV detection methods [34-36]. Our study was not designed to confirm the association between HPV positive HNSCC tumors and improved survival, and consequently, did not take into account other factors influencing prognosis. Therefore, no conclusions regarding HPV positive HNSCC tumors and overall survival can be made. Furthermore, the value of cetuximab in HPV-related HNSCC is unclear. Recent data challenge the role of cetuximab therapy in HPV positive oral squamous cell carcinoma [37,38]. Therefore, the role of cetuximab in treatment of HPV-associated HNSCC needs to be explored in prospective clinical trials.

## Conclusion

To conclude, this retrospective study detected two silent *EGFR* tyrosine kinase mutations and no *EGFRvIII* mutations. Due to discrepancy between HRMA and sequencing results, *KRAS* mutation frequency varies from 0.0% to 7.0%. Although the mutation status of *EGFR* and *KRAS* are used as predicting biomarkers for EGFR targeted therapy in other tumor types, their (extremely) low prevalence in HNSCC will likely preclude a major role in helping to define treatment options in these patients. Therefore, other genes are likely involved in resistance to anti-EGFR therapy in HNSCC and more in-depth-studies are needed to unravel these mechanisms.

## Abbreviations

EGFR: Epidermal growth factor receptor; HNSCC: Head and neck squamous cell carcinoma; HRMA: High resolution melting analysis; mCRC: Metastatic colorectal cancer; TKIs: Tyrosine kinase inhibitors; NSCLC: Non-small cell lung cancer; HPV: Human papilloma virus; FFPE: Formalin-fixed paraffin embedded.

## Competing interests

Jan B. Vermorken: Merck-Serano (consulting/advisory relationship, honoraria, travel compensation); Bristol-Myers Squibb (honoraria, travel compensation); Lilly (travel compensation). Marc Peeters: Amgen/Merck-Serono (consulting/advisory relationship, research funding, honoraria). The other authors declare that they have no competing interests.

## Authors’ contributions

CB carried out the KRAS mutation study, performed the statistical analysis and drafted the manuscript. CW and PP carried out the EGFR tyrosine kinase mutation study. IVB and MK carried out the EGFRvIII mutation study. MB participated in the design and coordination of the study, carried out the HPV detection and helped to draft the manuscript. DvD, CVL and PP provided clinical samples and clinicopathological data. VD, AW, PS, MP and FL participated in the design and coordination of the study. All authors read and approved the final manuscript.
